# Comparative serum metabolomic profiling of hypertension in different traditional Chinese medicine syndromes

**DOI:** 10.3389/fmolb.2025.1655493

**Published:** 2025-12-03

**Authors:** Sunan Yong, Le Shao, Zhi Liu, Chi Fang, Xiaobing Xie, Su Li

**Affiliations:** The First Clinical College of Traditional Chinese Medicine, Hunan University of Chinese Medicine, Changsha, Hunan, China

**Keywords:** serum, metabolome, hypertension, traditional Chinese medicine syndromes, WGCNA

## Abstract

**Background:**

Hypertension is a crucial risk factor for cardiovascular diseases. Previous studies have revealed the serum metabolic profiles of patients with hypertension, laying the groundwork for accurate diagnosis and potential therapeutic target identification. While hypertension has well-documented biochemical signatures, TCM classifies it into distinct syndromes based on patterns of clinical manifestations and underlying pathophysiological concepts. From the perspective of Traditional Chinese Medicine (TCM), hypertension can be classified into several distinct syndromes, including Liver Yang Rising (LYR), Internal Phlegm-Dampness Accumulation (IPDA), and Liver-Kidney Yin Deficiency (LKYD). The present study aimed to identify the metabolic biomarkers for TCM syndromes by metabolomic analysis.

**Methods:**

Metabolomic profiling of LYR, IPDA, and LKYD was performed (10 cases per group, sampled randomly) among thirty hypertensive patients and ten healthy controls recruited from the First Hospital of Hunan University of Chinese Medicine, Changsha, China. Hypertension and TCM syndrome classification were confirmed by clinicians, and participants with severe organ dysfunction or acute illnesses were excluded. Ultra-performance liquid chromatography-tandem mass spectrometry was used to identify differentially expressed metabolites (DEMs), weighted correlation network analysis (WGCNA) was applied to construct syndrome-associated networks, and multivariate ROC-based exploratory analysis identified key biomarkers with high diagnostic performance.

**Results:**

Thirty-seven DEMs were identified for each syndrome comparison (LYR vs. control, IPDA vs. control, and LKYD vs. control), with 26 shared DEMs enriched in multiple metabolic pathways. Pairwise comparisons among TCM syndromes revealed distinct metabolic profiles, including glycated amino acids and tryptophan derivatives. WGCNA identified hub metabolites such as p-Xylene and Octinoxate. Multivariate ROC analysis yielded ten biomarkers with high diagnostic accuracy (AUC = 0.944), offering potential for distinguishing TCM-based hypertension subtypes and guiding targeted interventions.

**Conclusion:**

Distinct metabolic signatures of TCM-based hypertension syndromes were identified, along with serum biomarkers showing high diagnostic accuracy. These findings support more precise syndrome differentiation and offer potential targets for personalized hypertension management.

## 1 Introduction

Traditional Chinese Medicine (TCM) provides a comprehensive framework for understanding hypertension through the lens of systemic imbalances and syndrome differentiation (Wang and Xiong, 2013). Rather than focusing solely on blood pressure levels, TCM classifies hypertension into distinct patterns such as Liver Yang Rising (LYR), Internal Phlegm-Dampness Accumulation (IPDA), and Liver-Kidney Yin Deficiency (LKYD) (Wang et al., 2014). This syndrome-based approach enables individualized treatment strategies that integrate herbal medicine, acupuncture, and lifestyle modification (Zewei et al., 2023). The incorporation of TCM into modern clinical practice offers potential for more personalized and holistic management of hypertension.

Although hypertension is well characterized by biochemical markers, its clinical presentation can vary widely among patients. In TCM, these variations are interpreted as distinct syndromes, classified according to patterns of symptoms and underlying pathophysiological principles. While hypertension has well-documented biochemical signatures, TCM classifies it into distinct syndromes based on patterns of clinical manifestations and underlying pathophysiological concepts. In TCM, hypertension is regarded as a manifestation of underlying imbalances within the body’s vital substances—Qi, Blood, Yin, and Yang—and their associated organ systems, particularly the liver, kidney, and spleen (Qu et al., 2022). The advantages of TCM lies on: holistic approach and individualized treatment, addressing both symptoms and root causes, fewer side effects and suitability for long-term use, emphasis on lifestyle and preventive care as well as integration of emotional and psychological health (Zhang et al., 2024). Nevertheless, a systematic understanding of hypertension subtypes within the framework of TCM remains insufficient, which poses a significant challenge to the advancement of TCM approaches in the prevention and management of hypertension (Wang et al., 2022).

TCM considers hypertension to fall under the categories of “dizziness,” “headache,” and “palpitations,” which are understood through the theory of syndrome differentiation. The pathogenesis is believed to involve internal wind, phlegm, fire, or deficiency, resulting from emotional stress, improper diet, aging, or constitutional weakness (Wang et al., 2014). Treatment focuses on restoring internal balance through herbal medicine, acupuncture, dietary regulation, and lifestyle adjustment, aiming to address the root cause rather than just the symptoms (Zhang et al., 2021). A clear identification of specific syndromes is of great significance for an accurate understanding of these classifications and for the development of individualized therapeutic approaches. Serum metabolic analysis plays a critical role in understanding the pathophysiology, diagnosis, and management of hypertension (Xu et al., 2025). By profiling metabolites in the blood, researchers and clinicians can identify specific biochemical changes associated with elevated blood pressure (Kanbay et al., 2023). This approach provides insights into metabolic pathways that may contribute to the development and progression of hypertension, such as lipid metabolism, glucose regulation, amino acid pathways, and oxidative stress (Tian and Liang, 2021). Moreover, serum metabolomics can help uncover early biomarkers of hypertension before clinical symptoms appear, enabling timely intervention and personalized treatment strategies (Zhao et al., 2023). It also offers a deeper understanding of the heterogeneity of hypertension, facilitating classification of patients based on metabolic phenotypes (Litwin and Kułaga, 2021).

Currently, there is a paucity of data regarding the blood metabolomic signatures corresponding to various TCM syndromes of hypertension. Elucidating these profiles would improve the metabolic understanding of TCM syndrome classification and play a crucial role in advancing personalized treatment strategies and fostering the integration of TCM with modern medical practices. This study focuses on investigating the serum metabolomic differences among patients with different TCM syndromes of hypertension—LYR, IPDA, and LKYD. By identifying key differential metabolites, the study aims to establish a relationship between TCM syndrome classification and serum metabolic profiles. The findings of this research may not only facilitate the screening of potential diagnostic biomarkers, but also provide valuable insights into the distinct pathophysiological characteristics of various TCM syndromes of hypertension.

## 2 Materials and methods

### 2.1 Patients

Thirty patients with hypertension and ten healthy individuals were enrolled in this study. All participants were recruited from the First Hospital of Hunan University of Chinese Medicine, Changsha, China (Ethical code: HN-LL-LW-2025-023). The basic information of the samples is listed in Supplementary Table S1. Eligible participants were patients with a confirmed diagnosis of hypertension and classified as LYR, IPDA, or LKYD by clinicians at the First Hospital of Hunan University of Chinese Medicine according to the Expert Consensus on Diagnosis and Treatment of Hypertension with Traditional Chinese Medicine (Society of Cardiovascular Diseases, 2019); individuals who, in addition to hypertension, presented with severe functional impairment of major organs (heart, liver, kidneys, or lungs) or with acute or critical illnesses such as malignant neoplasms were excluded from the study. Control participants had no history of cardiovascular or systemic disease, no significant abnormalities on clinical examination, and did not meet diagnostic criteria for hypertension (Supplementary Figure S1). Clinicians at the First Hospital of Hunan University of Chinese Medicine confirmed the participants’ health status. The present study complied with the Declaration of Helsinki.

### 2.2 Sample collection and preprocessing procedures

To identify biomarker for the specific syndromes of hypertension, venous blood samples were collected from all participants, and serum was isolated for metabolomic analysis. Non-targeted metabolomics analysis was performed using ultra-performance liquid chromatography-tandem mass spectrometry (UPLC-MS/MS) by Shenzhen Wininnovate BIO Co., Ltd. (Shenzhen, China). Metabolites were extracted from serum samples (50 μL per sample) using a 50% methanol-water solution. Subsequently, 100 μL of the metabolite extract was mixed with 400 μL of pre-chilled 50% methanol-acetonitrile solution and subjected to ultrasonication for 10 min. The mixture was incubated at −20 °C for 1 h, followed by centrifugation at 20,000 × g for 15 min at 4 °C. The supernatant was collected and dried to completeness overnight under vacuum. The dried residue was reconstituted in a 50% acetonitrile-water solution, sonicated for 10 min, and centrifuged again at 20,000 × g for 15 min at 4 °C. The resulting supernatant was transferred to vials for analysis. Quality control (QC) samples were prepared by pooling 10 μL aliquots from each sample and processed identically.

### 2.3 Metabolite profiling and annotation

Non-targeted metabolomics analysis was performed using an UltiMate 3000 ultra-performance liquid chromatography (UPLC) system coupled with a Q Exactive Plus high-resolution tandem mass spectrometer (Thermo Fisher Scientific, United States) at Shenzhen Wininnovate BIO Co., Ltd. Serum metabolites were extracted and analyzed in both positive and negative ion modes. Chromatographic separation was achieved on an ACQUITY UPLC HSS T3 column (100 mm × 2.1 mm, 1.7 μm, Waters, United States) at 50 °C, with a mobile phase of 0.1% formic acid in water (A) and 0.1% formic acid in acetonitrile (B) and a standard gradient at a flow rate of 0.3 mL min^−1^.

Raw data were processed using XCMS (https://xcmsonline.scripps.edu) for peak detection and alignment. Metabolites were annotated by matching accurate mass (m/z) and retention time to entries in the Human Metabolome Database (HMDB, version 4.0) and the Kyoto Encyclopedia of Genes and Genomes (KEGG), with a mass tolerance of 10 ppm. Candidate identifications were further supported by isotopic distribution analysis and comparison of MS/MS fragments with composite libraries (LipidBlast v37, MassBank, HMDB v4.0, and an in-house spectrum database). Detailed instrumental settings (e.g., voltages, gas pressures, injection times, AGC targets) are provided in the Supplementary Methods.

### 2.4 Screening and analysis of differentially expressed metabolites

Metabolomics data were subjected to preprocessing, including logarithmic transformation and z-score standardization. Missing value imputation was performed using half-minimum substitution. Principal component analysis (PCA) and orthogonal partial least-squares discriminant analysis (OPLS-DA) were employed to explore data structure and discriminate between groups. Differentially expressed metabolites (DEMs) were identified based on a significance threshold of *P* < 0.05, adjusted for multiple comparisons using the Benjamini–Hochberg false discovery rate (FDR) correction, and a variable importance in projection (VIP) score >1. Annotated metabolites, identified by matching against the Kyoto Encyclopedia of Genes and Genomes (KEGG) Compound database (https://www.genome.jp/kegg/compound/), were mapped to metabolic pathways using the KEGG Pathway database (https://www.genome.jp/kegg/pathway.html; Release 102.0). Pathway enrichment analysis was conducted using a hypergeometric distribution, with pathways deemed significantly enriched at *P* < 0.05.

### 2.5 Construction of weighted gene co-expression network for metabolites

Weighted gene co-expression network analysis (WGCNA) was employed to delineate significant metabolic modules and central metabolites. The analysis was conducted using the WGCNA R package. All the metabolites detected in the present study were included in the WGCNA analysis (716 metabolites). A hierarchical clustering dendrogram was generated through a dynamic tree-cutting algorithm, as described in previous studies (Langfelder and Horvath, 2008; Zhou et al., 2020). Associations between metabolic modules and TCM syndromes were assessed to identify modules with the strongest phenotypic correlations. These modules were selected for subsequent functional enrichment analysis. A network visualization of key modules was constructed using Cytoscape software (version 3.7.2; Shannon et al., 2003). Hub metabolites within these modules were determined using the maximal clique centrality (MCC) algorithm implemented in the Cytohubba plug-in for Cytoscape, with the top five metabolites designated as hub metabolites.

### 2.6 Multivariate ROC curve analysis

This study analyzed data from 40 participants (10 with normal blood pressure and 30 with hypertension, categorized into liver yang hyperactivity, phlegm-heat disturbance, and liver-kidney yin deficiency groups) using MetaboAnalyst 6.0 (https://www.metaboanalyst.ca) for multivariate Receiver Operating Characteristic (ROC) analysis. Data were auto-scaled in MetaboAnalyst, and discriminatory performance was assessed using Partial Least Squares-Discriminant Analysis (PLS-DA) and Random Forest (RF) models. Model accuracy was evaluated through 10-fold cross-validation and 1,000 bootstrap iterations to estimate AUC, sensitivity, and specificity, and optimal thresholds were determined using Youden’s index.

## 3 Results

### 3.1 Metabolites identification

**FIGURE 1:**
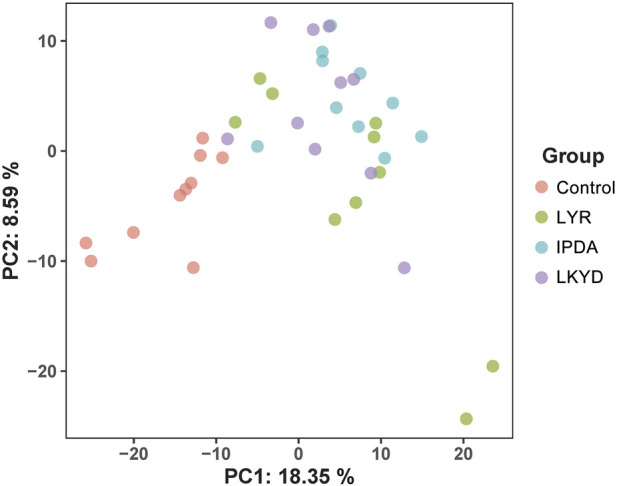
Principal component analysis (PCA) scores plot of metabolome distribution of the hypertensive patients and TCM syndromes. Liver Yang Rising: LYR; Internal Phlegm-Dampness Accumulation: IPDA; Liver-Kidney Yin Deficiency: LKYD.

A total of 716 metabolites were identified (Supplementary Table S2) assigned into 14 super classes (Supplementary Table S2). The PCA showed that the control group were separated from LYR, IPDA and LKYD groups while the groups of the hypertension could not be separated (Figure 1). A total of 92.88% of the metabolites in the QC samples exhibited a relative standard deviation (RSD) of less than 15%, demonstrating the high analytical precision and reproducibility of the metabolomics platform, and confirming the reliability of the data for subsequent analysis (Supplementary Table S2).

### 3.2 Hypertension alters metabolites in serum

**FIGURE 2:**
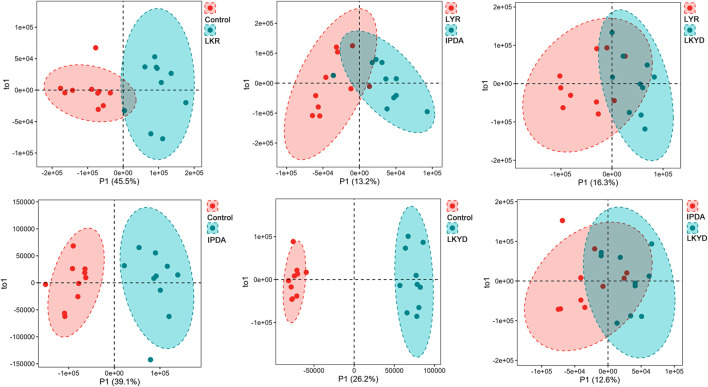
Orthogonal partial least square discriminant (OPLS-DA) analysis of metabolites. Liver Yang Rising: LYR; Internal Phlegm-Dampness Accumulation: IPDA; Liver-Kidney Yin Deficiency: LKYD.

**FIGURE 3:**
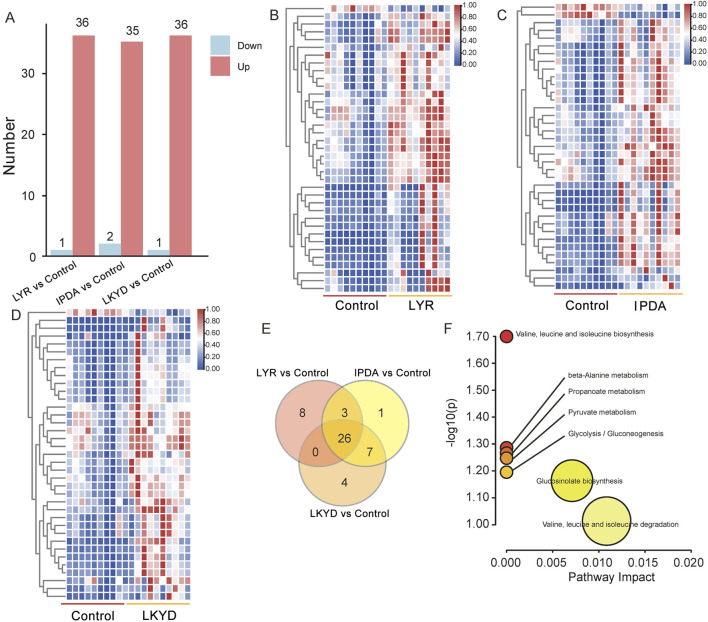
Differentially expressed metabolites (DEMs) compared by TCM syndromes and control. **(A)** DEMs between the TCM syndromes and control group. **(B)** Heatmap of DEMs between the control and Liver Yang Rising (LYR). **(C)** Heatmap of DEMs between the control and Internal Phlegm-Dampness Accumulation (IPDA). **(D)** Heatmap of DEMs between the control and Liver-Kidney Yin Deficiency (LKYD). **(E)** Venn diagram of DEMs from the three comparisons. **(F)** KEGG enrichment analysis of the 26 DEMs overlapped from the three comparisons.

OPLS-DA demonstrated clear separation between groups (Figure 2). The OPLS-DA model exhibited high R2X, R2Y, and Q2 values, indicating strong explanatory power and predictive capability. These results confirmed that the OPLS-DA models constructed under both positive and negative ionization modes were robust and not overfitted. First, we compared the hypertension groups with the control group. The LYR vs. control comparison identified 37 differentially expressed metabolites (DEMs), including 1 downregulated and 36 upregulated in the hypertension group relative to control (Figures 3A,B). For IPDA vs. control, 2 downregulated and 35 upregulated metabolites were identified (Figures 3A,C). The LKYD vs. control comparison revealed 1 downregulated and 36 upregulated metabolites (Figures 3A,D). A total of 26 metabolites (70.27% of those identified) were shared across all three comparisons, indicating high concordance among the hypertension groups relative to the control (Figure 3E; Supplementary Table S3). KEGG enrichment analysis showed that these metabolites were involved in valine, leucine, and isoleucine degradation, glucosinolate biosynthesis, glycolysis/gluconeogenesis, pyruvate metabolism, propanoate metabolism, beta-alanine metabolism, and valine, leucine, and isoleucine biosynthesis pathways (Figure 3F).

### 3.3 Identification of differentially expressed metabolites (DEMs) in TCM syndromes

**FIGURE 4:**
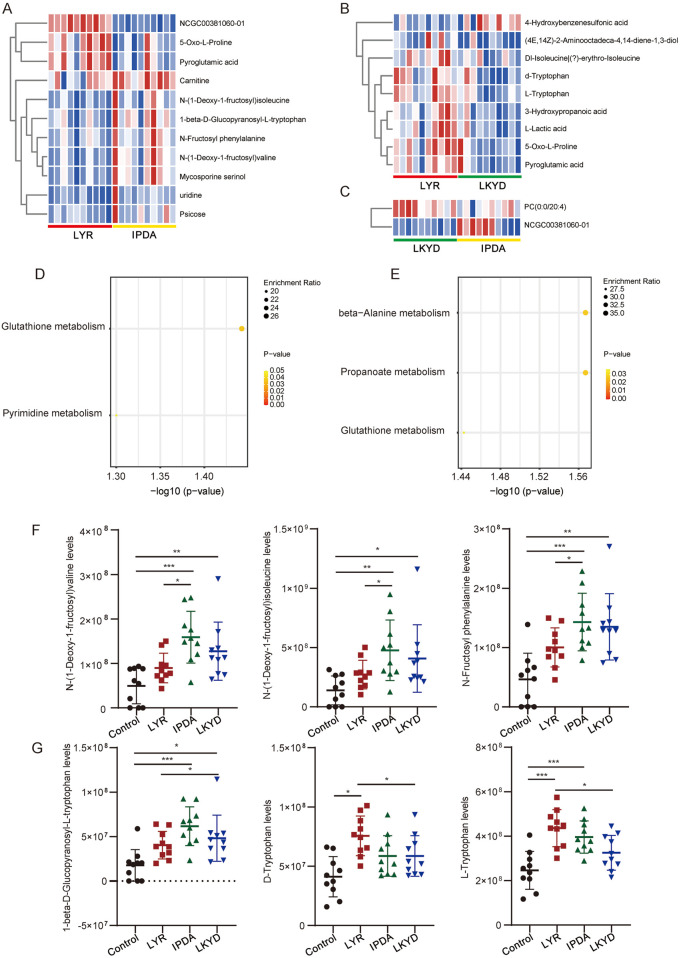
Differentially expressed metabolites (DEMs) compared by TCM syndromes pairwise. **(A)** Heatmap of DEMs between Liver Yang Rising (LYR) and Internal Phlegm-Dampness Accumulation (IPDA). **(B)** Heatmap of DEMs between Liver Yang Rising (LYR) and Liver-Kidney Yin Deficiency (LKYD). **(C)** Heatmap of DEMs between Liver-Kidney Yin Deficiency (LKYD) and Internal Phlegm-Dampness Accumulation (IPDA). **(D)** KEGG enrichment analysis of the DEMs from Liver Yang Rising (LYR) and Internal Phlegm-Dampness Accumulation (IPDA) comparison. **(E)** KEGG enrichment analysis of the DEMs from Liver Yang Rising (LYR) and Liver-Kidney Yin Deficiency (LKYD). **(F)** Glycated amino acids levels in the tested groups. *, *P* < 0.05; **, *P* < 0.01; ***, *P* < 0.001. **(G)** Tryptophan levels in the tested groups. *, *P* < 0.05; **, *P* < 0.01; ***, *P* < 0.001.

Furthermore, we compared the hypertension groups pairwise. The LYR vs. IPDA comparison identified 11 metabolites (3 upregulated and 8 downregulated), LYR vs. LKYD revealed 9 metabolites (8 upregulated and 1 downregulated), and LKYD vs. IPDA showed 2 metabolites (1 up- and 1 downregulated) (Figures 4A–C; Supplementary Table S4). KEGG enrichment analysis indicated that the LYR vs. IPDA metabolites were involved in glutathione and pyrimidine metabolism (Figure 4D), while those from LYR vs. LKYD were enriched in beta-alanine metabolism, propanoate metabolism, and glutathione metabolism (Figure 4E). From the LYR vs. IPDA comparison, three glycated amino acids including N-(1-Deoxy-1-fructosyl) valine, N-(1-Deoxy-1-fructosyl) isoleucine, and N-Fructosyl phenylalanine had significantly higher levels in IPDA group compared to LYR. The glycated amino acids were also increased in hypertension groups comparted to the control (Figure 4F). By comparing the LYR vs. LKYD group, the 1-beta-D-Glucopyranosyl-L-tryptophan, D-Tryptophan, and L-Tryptophan are Tryptophan-based modifiers which were significantly changed (Figure 4G).

### 3.4 Hub metabolites in TCM syndromes

**FIGURE 5:**
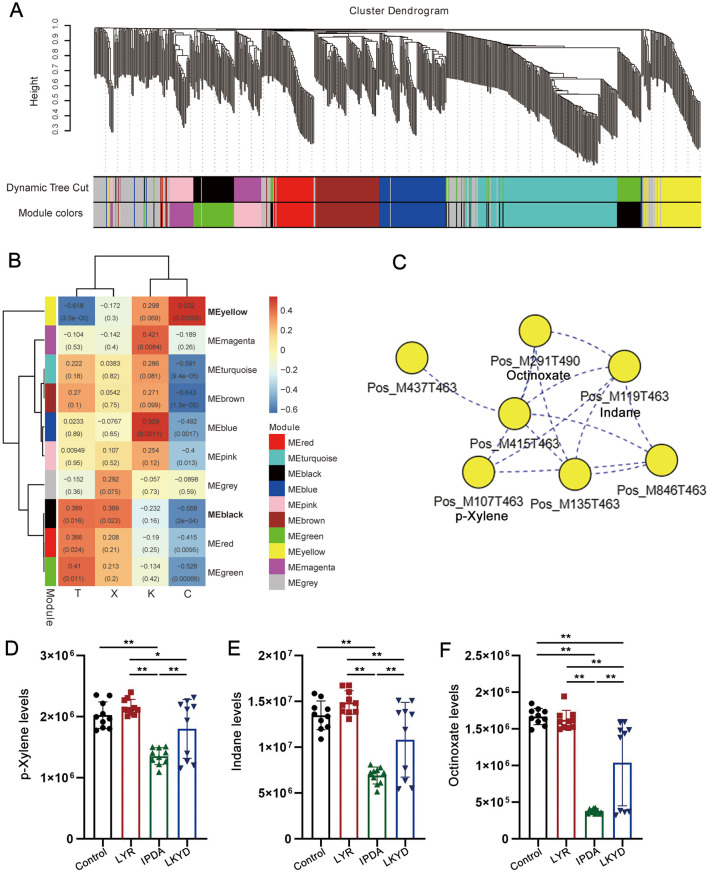
WGCNA and identification of hub metabolites from the TCM syndromes. **(A)** Clustering dendrogram of metabolites with dissimilarity based on topological overlap, together with assigned module colors. **(B)** Heatmap illustrating the correlation between modules and metabolites. The numbers in the box showed Pearson correlation coefficient (r) and the *P* values were shown in the brackets. **(C)** Key network with hub metabolites from yellow module. **(D)** p-Xylene levels in the tested groups. *, *P* < 0.05; **, *P* < 0.01; ***, *P* < 0.001. **(E)** Indane levels in the tested groups. *, *P* < 0.05; **, *P* < 0.01; ***, *P* < 0.001. **(F)** Octinoxate levels in the tested groups. *, *P* < 0.05; **, *P* < 0.01; ***, *P* < 0.001.

By WGCNA, 10 modules were identified (Figure 5A). The correlation analysis indicated that of the ten modules, nine demonstrated significant correlations (*P* < 0.05) with at least one phenotypic trait group (Figure 5B). The yellow and black modules were significantly correlated with three groups (*P* < 0.05). We then scanned the correlations among metabolites to construct a network for the modules (Figure 5C). A key network from yellow module were constructed contained 7 metabolites. p-Xylene, Indane, and Octinoxate showed lowest levels in IPDA, intermediate in LKYD, and highest in Control and LYR (Figures 5D,E) (*P* > 0.05). All the three compounds showed the lowest levels in IPDA compared to other groups and the highest levels were found in control and LYR groups while LKYD had the medium levels of these compounds (Figures 5D–F) (*P* < 0.05).

### 3.5 Identification of biomarkers for syndromes

**FIGURE 6:**
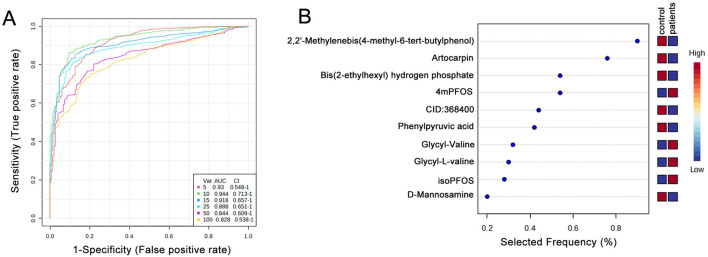
Biomarker identification and prediction through multivariate ROC curve-based exploratory analysis. **(A)** Summary of ROC curves generated by MetaboAnalyst from six biomarker models constructed using varying numbers of features (5, 10, 15, 25, 50, and 100). Each curve is accompanied by its corresponding AUC value and confidence interval, providing an assessment of model performance. **(B)** The top 10 candidate biomarkers were identified based on their selection frequency during cross-validation, indicating their potential relevance and robustness in distinguishing between groups.

Potential biomarkers were identified, and their diagnostic accuracy was evaluated using receiver operating characteristic (ROC) analysis. Partial least squares discriminant analysis (PLS-DA) was applied as a classification and feature selection method to conduct a multivariate exploratory ROC analysis. The top ten features yielded an area under the ROC curve (AUC) of 0.944 (95% CI: 0.713–1) (Figure 6A). These ten biomarkers associated with the TCM syndromes included 2,2′-Methylenebis (4-methyl-6-tert-butylphenol), Artocarpin, Bis(2-ethylhexyl) hydrogen phosphate, 4mPFOS, CID:368,400, Phenylpyruvic acid, Glycyl-Valine, Glycyl-L-valine, isoPFOS, and D-Mannosamine (Figure 6B; Supplementary Tables S6, S7).

## 4 Discussion

In this study, we compared serum metabolic profiles among hypertensive patients with different TCM syndromes and healthy controls. Our results demonstrated that hypertension significantly alters the serum metabolic profile, indicating widespread disruptions in biochemical pathways. Notably, distinct sets of DEMs were identified across the TCM syndromes, suggesting syndrome-specific metabolic heterogeneity. The present findings provide novel insights into the metabolic basis of hypertension from both biomedical and TCM perspectives, and offer potential avenues for objective syndrome classification and personalized treatment strategies.

Metabolomics studies have consistently revealed significant differences in the concentrations of various metabolites in hypertensive individuals compared to controls. For example, essential hypertension is associated with elevated levels of arginine and reduced levels of alanine, pyruvate, methionine, adenine, and uracil in serum, suggesting disruptions in amino acid and nucleotide metabolism (Ameta et al., 2017). In addition, increases in lipid-related metabolites such as VLDL, LDL, lactic acid, and acetone have been observed, indicating dysregulated lipid and energy metabolism (Hu et al., 2023). These serum metabolites often precede the clinical onset of elevated blood pressure, highlighting their potential as early biomarkers. Furthermore, changes in ceramides, glycerolipids, and phosphatidylcholines have been linked to both diastolic and systolic blood pressure progression, implicating lipid metabolism in the pathogenesis of hypertension (Lin et al., 2020; Niu et al., 2022). These findings underscore the utility of serum metabolomic profiling in identifying early metabolic signatures associated with hypertension, enabling improved risk stratification and potential therapeutic targeting. In the present study, we found that the vast majority of differentially expressed metabolites (DEMs) were upregulated in hypertensive cases. Similar to the previous reports, metabolites in amino acid and nucleotide metabolism, pyruvate metabolism, and Valine, leucine and isoleucine biosynthesis pathways were found significantly changed in hypertensive individuals (Seryapina et al., 2023). We also acknowledge that the normotensive group in our study was younger than the hypertensive group, and therefore age differences may have contributed to some of the observed changes. Nevertheless, our findings remain reliable for two reasons: first, the overall results are consistent with previous reports (Lin et al., 2020; Niu et al., 2022; Seryapina et al., 2023); and second, the overlap between the DEMs identified in this study and those previously reported as aging-related is limited (Balashova et al., 2022). Taken together, this suggests that the DEMs identified here are likely to be robust and hypertension-related. These metabolites hold significant predictive value for incident hypertension, particularly when analyzed alongside established risk factors.

TCM classifies hypertension into different syndrome types, such as liver yang hyperactivity, yin deficiency with yang hyperactivity, dampness-phlegm accumulation, and deficiency of both yin and yang (Liu, 2009). Recent advances in metabolomics provide a powerful platform to explore the biological basis of these syndromes by profiling serum metabolites (Chapinal et al., 2011; Yu et al., 2011). Studies have shown that each TCM syndrome type in hypertensive patients exhibits distinct metabolic features. For example, liver yang hyperactivity is often associated with disturbances in amino acid and arachidonic acid metabolism, while the dampness-phlegm syndrome is linked with elevated uric acid and disrupted lipid and glucose metabolism (Yang and Lao, 2019). Though traditional statistical approaches like PCA and PLS-DA are useful for distinguishing hypertensive patients from healthy individuals, they are less effective in separating the nuanced TCM syndromes. Mahalanobis distance analysis has demonstrated better performance in differentiating these subtypes (Lu et al., 2007). These findings suggest that integrating metabolomics into TCM can enhance the scientific understanding of syndrome differentiation, supporting more personalized and precise treatment strategies for hypertension (Nikolic et al., 2014; Onuh and Aliani, 2020). Our present study also showed that several DEMs existed in the different syndrome types which is in accordance with the previous researches. From the present results, we found that the glutathione metabolism and pyrimidine metabolism were different among the LYR vs. IPDA. Depletion of reduced glutathione (GSH) and alterations in the activity of related enzymes such as glutathione peroxidase (GPx) and glutathione reductase (GR) have been observed in both animal models and human subjects with hypertension (Rybka et al., 2011). Such imbalances enhance reactive oxygen species (ROS) accumulation, leading to increased vascular tone, inflammation, and remodeling of the arterial wall (Hirooka et al., 2011). On the other hand, pyrimidine metabolism, essential for DNA and RNA synthesis, has been associated with cell proliferation, vascular repair, and immune response in hypertensive states (Devlin et al., 2000). The LYR vs. LKYD comparison indicated the changes of metabolites in beta-Alanine metabolism, propanoate metabolism, and glutathione metabolism. Beta-alanine metabolism contributes to the synthesis of carnosine, a dipeptide with antioxidant and pH-buffering properties (Kojima, 2024). Altered beta-alanine levels may affect vascular tone and oxidative balance, as carnosine helps scavenge reactive oxygen species (ROS) and chelate metal ions, thereby potentially mitigating oxidative stress-induced vascular damage in hypertensive individuals (Meftahi and Jahromi, 2023). Propanoate metabolism, a component of short-chain fatty acid (SCFA) metabolism, has been increasingly recognized for its role in modulating gut microbiota-host interactions, inflammation, and vascular homeostasis (Das et al., 2023). Dysregulation of propanoate and related SCFAs can influence blood pressure through mechanisms involving G-protein coupled receptors (e.g., GPR41/43), endothelial nitric oxide synthesis, and systemic inflammation (Xu et al., 2022). Glutathione metabolism, central to redox regulation, detoxification, and cellular defense, is frequently disrupted in hypertension (Griendling et al., 2021). Interestingly, we also found that three glycated amino acids were higher in IPDA than that in LYR and control groups. Glycated amino acids, as early precursors of advanced glycation end-products (AGEs), contribute to the pathogenesis of hypertension by promoting oxidative stress, endothelial dysfunction, and vascular inflammation (Cai et al., 2024). Their accumulation, driven by insulin resistance and metabolic imbalance, disrupts vascular homeostasis and exacerbates blood pressure elevation (Onuh and Aliani, 2020). In addition, Tryptophan-based modifiers were significantly changed when compared the LYR vs. LKYD group. Tryptophan metabolism influences hypertension through its three major pathways—kynurenine, serotonin, and indole—which produce metabolites like kynurenine, serotonin, and indoxyl sulfate that modulate vascular function, oxidative stress, and inflammation (Theiler-Schwetz et al., 2023). Dysregulation of these pathways, particularly the accumulation of uremic toxins in chronic kidney disease, is associated with increased blood pressure, while interventions like tryptophan supplementation or melatonin therapy may reprogram these pathways to prevent hypertension in animal models (Hsu and Tain, 2020). Therefore, from the perspective of TCM, hypertension is a heterogeneous condition with multiple pathological types. The findings of this study indicate that certain metabolite differences correspond to distinct TCM syndromes, suggesting that the etiology and pathophysiological characteristics of hypertension may vary across different TCM pattern classifications.

We employed WGCNA to identified networks that correlated with the phenotypes of different syndrome types. Two modules included the yellow and black modules were significantly correlated with three groups. Thereinto, yellow module showed significant positive correlation with the syndromes. Thus, the core network of yellow module was constructed. We found that p-Xylene, Indane and Octinoxate as small molecule compounds were hub metabolites in the core network of yellow module. It has been reported that p-Xylene as a volatile organic compounds was related to as associated with increased incidence of nonfatal cardio-cerebrovascular events in US adults showing a potential risk for hypertension (Jing et al., 2024). Indane is compound that regulates G protein coupled receptor (GPCR) activity to emphasis on their structure–activity relationships (Vilums et al., 2015) and several members from GPCR family have been used as target for curing hypertension (Brinks and Eckhart, 2010). Less results have been reported that Octinoxate is related to hypertension. As a potential thyroid hormone disruptor, Octinoxate may be related to the onset of hypertension (Cahova et al., 2023). These clues suggested that the networks composed of core metabolites such as organic compounds are involved in the development of different types of hypertensions.

Blood pressure fluctuations are commonly used to diagnose hypertension. However, numerous pathological changes often occur before blood pressure levels begin to rise significantly. Therefore, discovering novel biomarkers and thoroughly elucidating the underlying mechanisms that drive hypertension progression is essential for earlier diagnosis and intervention. We evaluated potential biomarkers for diagnosing both normotensive individuals and different syndrome types of hypertensions. Our analysis of 10 distinct metabolites yielded an impressive Area Under the Curve (AUC) value of 0.944. Multivariate ROC analysis identified ten candidate biomarkers with an apparently high diagnostic accuracy (AUC = 0.944) (95% CI: 0.713–1); however, given the small sample size (n = 40), these findings should be interpreted cautiously, and the potential risk of overfitting should be acknowledged when considering their utility in distinguishing TCM-based hypertension subtypes and guiding targeted interventions. In the future, further evaluation of the stability and clinical applicability of these candidate biomarkers will be required. The identified metabolites include compounds, perfluorooctane sulfonate, phenylpyruvic acid, di-peptides, and D-mannosamine. All of these specific metabolites have previously been implicated in and shown to participate in the pathogenesis and progression of cardiovascular diseases, suggesting their direct relevance to hypertension (Kifer et al., 2021; Zhang et al., 2022; Shen et al., 2023). This research highlights the promising role of these metabolites as early diagnostic indicators, potentially allowing for more timely and targeted therapeutic strategies to prevent or mitigate the adverse effects of hypertension (Supplementary Figure S2).

## 5 Strengths and limitations

This study identified specific metabolites that differentiate hypertension patients across distinct TCM syndromes, highlighting their potential as biomarkers for precise syndrome classification. These findings provide a foundation for developing metabolite-based tools to support accurate diagnosis and personalized treatment in TCM-guided management of hypertension. However, the small sample size (n = 40) limits statistical power and raises the risk of overfitting, requiring validation in larger independent cohorts. Some detected compounds (e.g., p-xylene, indane, octinoxate) are exogenous chemicals and may reflect environmental exposure or sample contamination rather than intrinsic metabolism. Further studies should include larger, longitudinal cohorts and functional experiments to validate these biomarkers and clarify their mechanistic roles in hypertension and TCM syndromes.

## 6 Conclusion

This study reveals distinct metabolic signatures of hypertension and its TCM syndromes, suggesting potential biomarkers for more precise classification and management. While preliminary, these findings contribute to bridging metabolomics with TCM-based diagnosis. Future large-scale and mechanistic studies are needed to validate these results and translate them into clinical applications.

## Data Availability

The original contributions presented in the study are included in the article/Supplementary Material, further inquiries can be directed to the corresponding authors.

## References

[B1] Ameta K. Gupta A. Kumar S. Sethi R. Kumar D. Mahdi A. A. (2017). Essential hypertension: a filtered serum based metabolomics study. Sci. Rep. 7 (1), 2153. 10.1038/s41598-017-02289-9 28526818 PMC5438387

[B2] Balashova E. E. Maslov D. L. Trifonova O. P. Lokhov P. G. Archakov A. I. (2022). Metabolome profiling in aging studies. Biology 11 (11), 1570. 10.3390/biology11111570 36358271 PMC9687709

[B3] Brinks H. L. Eckhart A. D. (2010). Regulation of GPCR signaling in hypertension. Biochimica Biophysica Acta (BBA) - Mol. Basis Dis. 1802 (12), 1268–1275. 10.1016/j.bbadis.2010.01.005 20060896 PMC2972307

[B4] Cahova J. Blahova J. Mares J. Hodkovicova N. Sauer P. Kroupova H. K. (2023). Octinoxate as a potential thyroid hormone disruptor – a combination of *in vivo* and *in vitro* data. Sci. Total Environ. 856, 159074. 10.1016/j.scitotenv.2022.159074 36181807

[B5] Cai S. Fu Y. Chen J. Tian M. Li X. (2024). Causal relationship between branched‐chain amino acids and hypertension: a mendelian randomization study. J. Am. Heart Assoc. 13 (5), e032084. 10.1161/JAHA.123.032084 38420789 PMC10944042

[B6] Chapinal N. Carson M. Duffield T. F. Capel M. Godden S. Overton M. (2011). The association of serum metabolites with clinical disease during the transition period. J. Dairy Sci. 94 (10), 4897–4903. 10.3168/jds.2010-4075 21943741

[B7] Das S. S. Kar A. Rajkumar S. Lee S. H. T. Alvarez M. Pietiläinen K. H. (2023). Cross-tissue single-nucleus RNA sequencing discovers tissue-resident adipocytes involved in propanoate metabolism in the human heart. Arteriosclerosis, Thrombosis, Vasc. Biol. 43 (10), 1788–1804. 10.1161/ATVBAHA.123.319358 37409528 PMC10538422

[B8] Devlin A. M. Clark J. S. Reid J. L. Dominiczak A. F. (2000). DNA synthesis and apoptosis in smooth muscle cells from a model of genetic hypertension. Hypertension 36 (1), 110–115. 10.1161/01.HYP.36.1.110 10904021

[B9] Griendling K. K. Camargo L. L. Rios F. J. Alves-Lopes R. Montezano A. C. Touyz R. M. (2021). Oxidative stress and hypertension. Circulation Res. 128 (7), 993–1020. 10.1161/CIRCRESAHA.121.318063 33793335 PMC8293920

[B10] Hirooka Y. Kishi T. Sakai K. Takeshita A. Sunagawa K. (2011). Imbalance of central nitric oxide and reactive oxygen species in the regulation of sympathetic activity and neural mechanisms of hypertension. Am. J. Physiology-Regulatory, Integr. Comp. Physiology 300 (4), R818–R826. 10.1152/ajpregu.00426.2010 21289238

[B11] Hsu C.-N. Tain Y.-L. (2020). Developmental programming and reprogramming of hypertension and kidney disease: impact of tryptophan metabolism. Int. J. Mol. Sci. 21 (22), 8705. 10.3390/ijms21228705 33218054 PMC7698939

[B12] Hu F. Yu H. Zong J. Xue J. Wen Z. Chen M. (2023). The impact of hypertension for metabolites in patients with acute coronary syndrome. Ann. Transl. Med. 11 (2), 50. 10.21037/atm-22-6409 36819519 PMC9929784

[B13] Jing L. Chen T. Yang Z. Dong W. (2024). Association of the blood levels of specific volatile organic compounds with nonfatal cardio-cerebrovascular events in US adults. BMC Public Health 24 (1), 616. 10.1186/s12889-024-18115-7 38408965 PMC10898104

[B14] Kanbay M. Copur S. Siriopol D. Yildiz A. B. Gaipov A. van Raalte D. H. (2023). Effect of tirzepatide on blood pressure and lipids: a meta-analysis of randomized controlled trials. Diabetes, Obes. Metabolism 25 (12), 3766–3778. 10.1111/dom.15272 37700437

[B15] Kifer D. Louca P. Cvetko A. Deriš H. Cindrić A. Grallert H. (2021). N-glycosylation of immunoglobulin G predicts incident hypertension. J. Hypertens. 39 (12), 2527–2533. 10.1097/HJH.0000000000002963 34285147 PMC7611954

[B16] Kojima C. (2024). Amino acid profiles associated with plant-rich protein diets May contribute to lower blood pressure. Hypertens. Res. 47 (10), 2964–2966. 10.1038/s41440-024-01849-7 39164419

[B17] Lin Y.-T. Salihovic S. Fall T. Hammar U. Ingelsson E. Ärnlöv J. (2020). Global plasma metabolomics to identify potential biomarkers of blood pressure progression. Arteriosclerosis, Thrombosis, Vasc. Biol. 40 (8), e227–e237. 10.1161/ATVBAHA.120.314356 32460578

[B18] Litwin M. Kułaga Z. (2021). Obesity, metabolic syndrome, and primary hypertension. Pediatr. Nephrol. 36 (4), 825–837. 10.1007/s00467-020-04579-3 32388582 PMC7910261

[B19] Liu Z. (2009). “Etiology in Chinese medicine,” in Essentials of Chinese medicine. Editor Liu Z. (London: Springer London), 131–164.

[B20] Lu Y.-h. Hao H.-p. Wang G.-j. Chen H.-x. Zhu X.-x. Xiang B.-r. (2007). Metabolomics approach to the biochemical differentiation of traditional Chinese medicine syndrome types of hypertension. Chin. J. Clin. Pharmacol. Ther. 12 (10), 1144.

[B21] Meftahi G. H. Jahromi G. P. (2023). Biochemical mechanisms of beneficial effects of beta-alanine supplements on cognition. Biochem. Mosc. 88 (8), 1181–1190. 10.1134/S0006297923080114 37758316

[B22] Nikolic S. B. Sharman J. E. Adams M. J. Edwards L. M. (2014). Metabolomics in hypertension. J. Hypertens. 32 (6), 1159–1169. 10.1097/HJH.0000000000000168 24675680

[B23] Niu Z. Wu Q. Luo Y. Wang D. Zheng H. Wu Y. (2022). Plasma lipidomic subclasses and risk of hypertension in middle-aged and elderly Chinese. Phenomics 2 (5), 283–294. 10.1007/s43657-022-00057-y 36939788 PMC9590468

[B24] Onuh J. O. Aliani M. (2020). Metabolomics profiling in hypertension and blood pressure regulation: a review. Clin. Hypertens. 26 (1), 23. 10.1186/s40885-020-00157-9 33292736 PMC7666763

[B25] Qu Y. Lv Y. Ji S. Ding L. Zhao F. Zhu Y. (2022). Effect of exposures to mixtures of lead and various metals on hypertension, pre-hypertension, and blood pressure: a cross-sectional study from the China national human biomonitoring. Environ. Pollut. 299, 118864. 10.1016/j.envpol.2022.118864 35063540

[B26] Rybka J. Kupczyk D. Kędziora-Kornatowska K. Motyl J. Czuczejko J. Szewczyk-Golec K. (2011). Glutathione-related antioxidant defense system in elderly patients treated for hypertension. Cardiovasc. Toxicol. 11 (1), 1–9. 10.1007/s12012-010-9096-5 21140238 PMC3035775

[B27] Seryapina A. Malyavko A. Polityko Y. Yanshole L. Tsentalovich Y. Markel A. (2023). Metabolic profile of blood serum in experimental arterial hypertension. Vavilov J. Genet. Breed. 27 (5), 530–538. 10.18699/VJGB-23-64 37867609 PMC10587007

[B28] Shen Y. Wang P. Yang X. Chen M. Dong Y. Li J. (2023). Untargeted metabolomics unravel serum metabolic alterations in smokers with hypertension. Front. Physiology 14, 1127294–2023. 10.3389/fphys.2023.1127294 36935758 PMC10018148

[B29] Society of Cardiovascular Diseases C. A. o.C. M. (2019). Expert consensus on diagnosis and treatment of hypertension with traditional Chinese medicine. Chin. J. Exp. Traditional Med. Formulae 25 (15), 217–221.

[B30] Theiler-Schwetz V. Trummer C. Grübler M. R. Keppel M. H. Zittermann A. Tomaschitz A. (2023). Associations of parameters of the tryptophan–kynurenine pathway with cardiovascular risk factors in hypertensive patients. Nutrients 15 (2), 256. 10.3390/nu15020256 36678127 PMC9862689

[B31] Tian Z. Liang M. (2021). Renal metabolism and hypertension. Nat. Commun. 12 (1), 963. 10.1038/s41467-021-21301-5 33574248 PMC7878744

[B32] Vilums M. Heuberger J. Heitman L. H. Ijzerman A. P. (2015). Indanes—Properties, preparation, and presence in ligands for G protein coupled receptors. Med. Res. Rev. 35 (6), 1097–1126. 10.1002/med.21352 26018667

[B33] Wang J. Xiong X. (2013). Evidence-Based Chinese medicine for hypertension. Evidence-Based Complementary Altern. Med. 2013 (1), 978398. 10.1155/2013/978398 23861720 PMC3686073

[B34] Wang J. Xiong X. Liu W. (2014). Traditional Chinese medicine syndromes for essential hypertension: a literature analysis of 13,272 patients. Evidence-Based Complementary Altern. Med. 2014 (1), 418206. 10.1155/2014/418206 24660016 PMC3934631

[B35] Wang Q. Lin J. Li C. Lin M. Zhang Q. Zhang X. (2022). Traditional Chinese medicine method of tonifying kidney for hypertension: clinical evidence and molecular mechanisms. Front. Cardiovasc. Med. 9, 1038480. 10.3389/fcvm.2022.1038480 36465462 PMC9709460

[B36] Xu J. Moore B. N. Pluznick J. L. (2022). Short-Chain fatty acid receptors and blood pressure regulation: council on hypertension Mid-Career award for research Excellence 2021. Hypertension 79 (10), 2127–2137. 10.1161/HYPERTENSIONAHA.122.18558 35912645 PMC9458621

[B37] Xu Y. Jiang Y. Lu J. Xu C. Li Q. Zhu H. (2025). Identification of serum metabolic markers in Non-obese hypertensive patients using non-targeted metabolomics. Sci. Rep. 15 (1), 18320. 10.1038/s41598-025-02162-0 40419532 PMC12106809

[B38] Yang M. Lao L. (2019). Emerging applications of metabolomics in traditional Chinese medicine treating hypertension: biomarkers, pathways and more. Front. Pharmacol. 10, 158–2019. 10.3389/fphar.2019.00158 30906260 PMC6418033

[B39] Yu Z. Kastenmüller G. He Y. Belcredi P. Möller G. Prehn C. (2011). Differences between human plasma and Serum metabolite profiles. PLOS ONE 6 (7), e21230. 10.1371/journal.pone.0021230 21760889 PMC3132215

[B40] Zewei Z. Fei Z. Chengwei Y. Bizhen G. Candong L. (2023). A Nomogram model for the early warning of essential hypertension risks based on the principles of traditional Chinese medicine syndrome elements. Digit. Chin. Med. 6 (3), 245–256. 10.1016/j.dcmed.2023.10.001

[B41] Zhang G.-X. Jin L. Jin H. Zheng G.-S. (2021). Influence of dietary components and traditional Chinese medicine on hypertension: a potential role for Gut Microbiota. Evidence-Based Complementary Altern. Med. 2021 (1), 5563073. 10.1155/2021/5563073 33986817 PMC8079198

[B42] Zhang Z. Wang F. Zhang Y. Yao J. Bi J. He J. (2022). Associations of serum PFOA and PFOS levels with incident hypertension risk and change of blood pressure levels. Environ. Res. 212, 113293. 10.1016/j.envres.2022.113293 35427595

[B43] Zhang H. Xia Z. Liu Y. Yu S. Shi H. Meng Y. (2024). Intervention of hypertension by acupuncture-related therapies: a network meta-analysis. Int. J. Older People Nurs. 19 (3), e12613. 10.1111/opn.12613 38701237

[B44] Zhao T. Yan Q. Wang C. Zeng J. Zhang R. Wang H. (2023). Identification of serum biomarkers of ischemic stroke in a hypertensive population based on metabolomics and lipidomics. Neuroscience 533, 22–35. 10.1016/j.neuroscience.2023.09.017 37806545

