# Supplementary figures and images for: Comparative serum metabolomic profiling of hypertension in different traditional Chinese medicine syndromes
Source: Front Mol Biosci. 2025 Dec 3;12:1655493. doi: 10.3389/fmolb.2025.1655493 (PMC12709170; doi:10.3389/fmolb.2025.1655493)

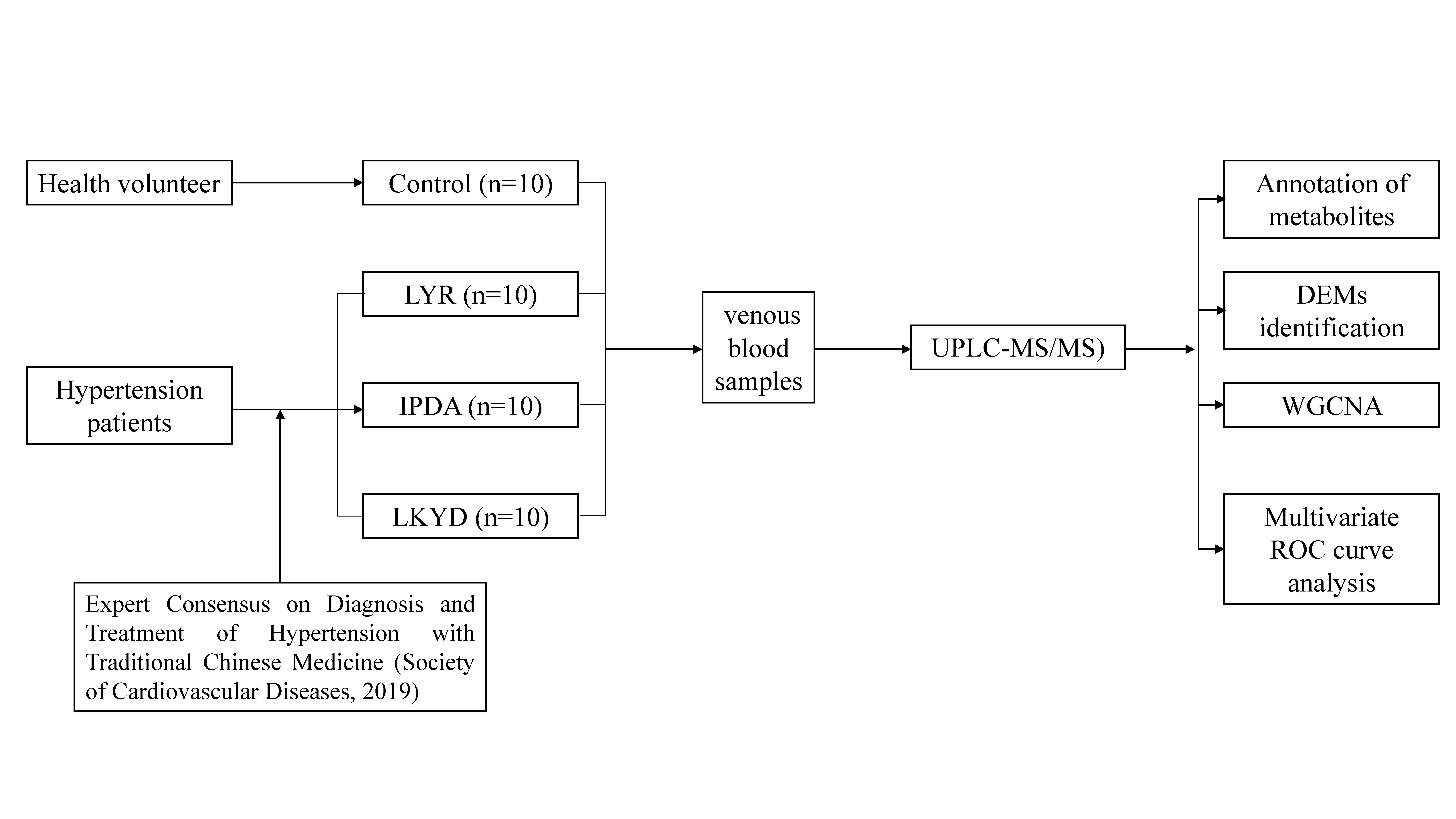

Supplement: Supplementary file 1 [file Image1.jpeg]

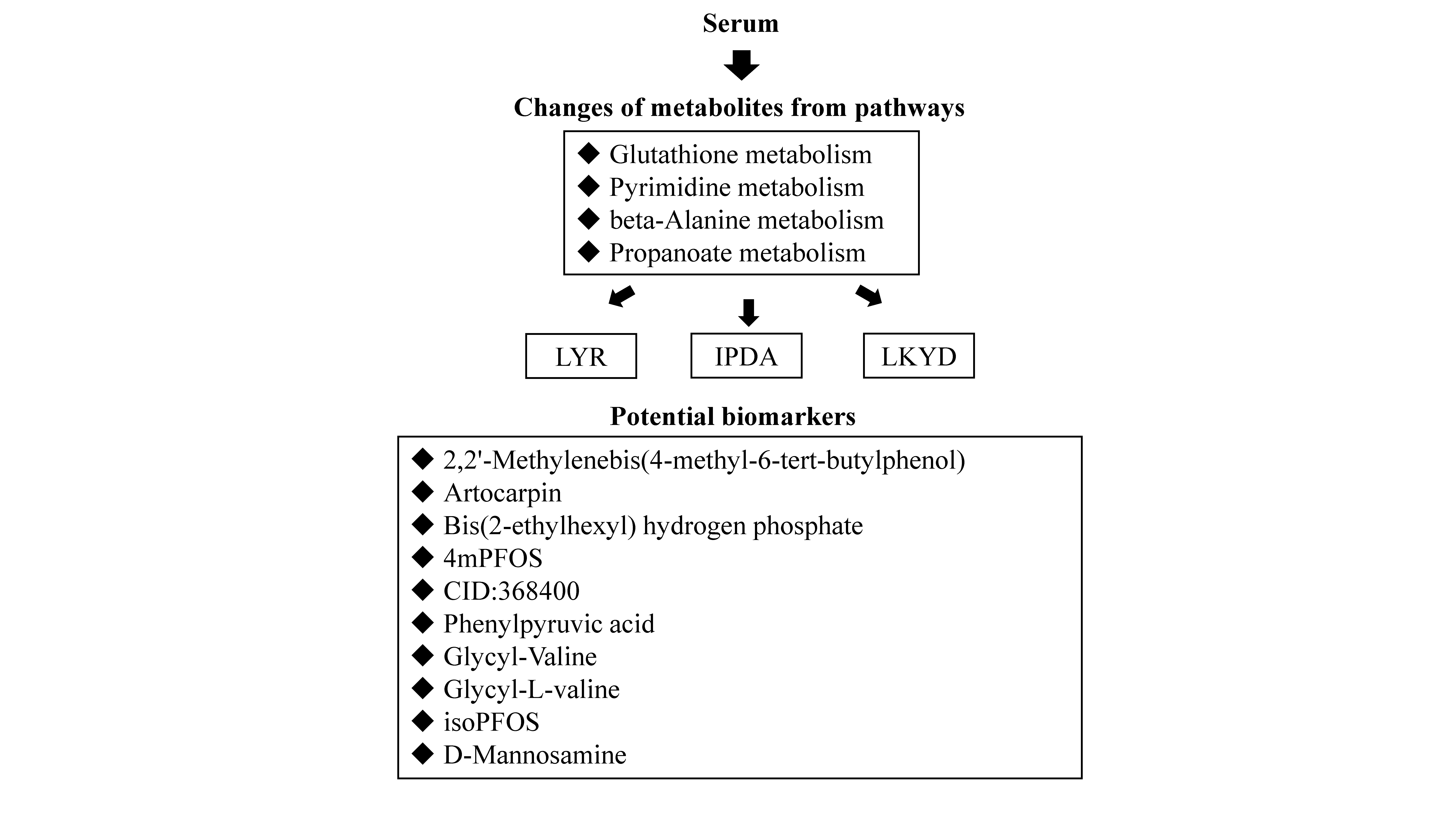

Supplement: Supplementary file 2 [file Image2.jpeg]
